# Image Morphing in Deep Feature Spaces: Theory and Applications

**DOI:** 10.1007/s10851-020-00974-5

**Published:** 2020-07-19

**Authors:** Alexander Effland, Erich Kobler, Thomas Pock, Marko Rajković, Martin Rumpf

**Affiliations:** 1 Institute of Computer Graphics and Vision, Graz University of Technology, Graz, Austria; 2 Institute of Numerical Simulation, University of Bonn, Bonn, Germany

**Keywords:** Image morphing, Metamorphosis model, Variational time discretization, Mosco convergence, Convolutional neural networks, 65D18, 37L65, 49M25, 53C22, 65L20

## Abstract

This paper combines image metamorphosis with deep features. To this end, images are considered as maps into a high-dimensional feature space and a structure-sensitive, anisotropic flow regularization is incorporated in the metamorphosis model proposed by Miller and Younes (Int J Comput Vis 41(1):61–84, 2001) and Trouvé and Younes (Found Comput Math 5(2):173–198, 2005). For this model, a variational time discretization of the Riemannian path energy is presented and the existence of discrete geodesic paths minimizing this energy is demonstrated. Furthermore, convergence of discrete geodesic paths to geodesic paths in the time continuous model is investigated. The spatial discretization is based on a finite difference approximation in image space and a stable spline approximation in deformation space; the fully discrete model is optimized using the iPALM algorithm. Numerical experiments indicate that the incorporation of semantic deep features is superior to intensity-based approaches.

## Introduction

In mathematical imaging, image morphing is the problem of computing a visually appealing transition of two images such that semantically corresponding regions are mapped onto each other. A well-known approach for image morphing is the metamorphosis model originally introduced by Miller et al. [16, 29, 30], which generalizes the flow of diffeomorphism model and the large deformation diffeomorphic metric mapping (LDDMM) which dates back to the pioneering work of Arnold [1] with its exploration and extension in imaging by Dupuis et al. [3, 10, 14, 18, 31, 32]. From the perspective of the flow of diffeomorphism model, each point of the reference image is transported to the target image in an energetically optimal way such that the image intensity is preserved along the trajectories of the pixels. Here, the energy measures the total dissipation of the underlying flow. The metamorphosis model additionally allows for image intensity modulations along the trajectories by incorporating the magnitude of these modulations, which is reflected by the integrated squared material derivative of image trajectories as a penalization term in the energy functional. Recently, the metamorphosis model has been extended to images on Hadamard manifolds [13, 20], to reproducing kernel Hilbert spaces [26], to functional shapes [6] and to discrete measures [25]. For a more detailed exposition of these models, we refer the reader to [17, 33] and the references therein.

Starting from the general framework for variational time discretization in geodesic calculus [27], a variational time discretization of the metamorphosis model for square-integrable images $L^2(\Omega ,\mathbb {R}^n)$ was proposed in [4]. Moreover, the existence of discrete geodesic paths as well as the Mosco convergence of the time discrete to the time continuous metamorphosis model was proven. However, the classical metamorphosis model, its time discrete counterpart and the spatial discretization based on finite elements in [4] exhibit several drawbacks:

- The comparison of images in their original gray- or color space is not invariant to natural radiometric transformations caused by lighting or material changes, shadows, etc., and hence might lead to a blending along the discrete geodesic path instead of flow-induced geometric transformations.
- Texture patterns, which are important for a natural appearance of images, are often destroyed along the geodesic path due to the color-based matching.
- Sharp interfaces such as object boundaries, which frequently coincide with depth discontinuities of a scene, are in general not preserved along a geodesic path because of the strong smoothness implied by the homogeneous and isotropic variational prior for the deformation fields.

To overcome these problems originating from the intensity-based matching, we propose a multiscale feature space approach incorporating the deep convolution neural network introduced in [28]. In detail, this convolutional neural network, which was trained to classify the ImageNet dataset [15], extracts semantic features using 19 weight layers, each composed of small $3\times 3$-convolution filters with subsequent nonlinear ReLU activation functions. This network defines a feature extraction operator, where each feature map is considered as a continuous map into some higher-dimensional feature space consisting of vectors in $\mathbb {R}^C$, where *C* ranges from 64 to 512 depending on the considered scale associated with a certain network layer. Throughout the paper, we refer to this network as VGG network (“Visual Geometry Group in Oxford”). Compared to the original time discrete metamorphosis model [4], we advocate a metamorphosis model in a deep feature space, which amounts to replacing the input images by feature vectors combining image intensities and semantic information generated by the feature extraction operator. To explicitly allow for discontinuities in the deformation fields, we introduce an anisotropic regularization of the time discrete deformation sequence. Since motion discontinuities and object interfaces in images commonly coincide, the considered anisotropy solely depends on the magnitude of image gradients.

We prove the existence of discrete geodesic paths for the deep feature metamorphosis model and discuss its Mosco convergence to the appropriate time continuous metamorphosis model in deep feature space. This in particular implies the convergence of time discrete to time continuous geodesic paths and establishes the existence of time continuous geodesics as minimizers of the time continuous metamorphosis model.

We propose a finite difference/third order B-spline discretization for the fully discrete feature space metamorphosis model and use the iPALM algorithm [24] for the optimization, which leads to an efficient and robust computation of morphing sequences that visually outperform the prior intensity-based finite element discretization discussed in [4]. This scheme is significantly less sensitive to intensity modulations due to the exploitation of semantic information.

Note that this publication is an extended version of the conference proceeding [12], in which the model is adapted and in addition a rigorous mathematical analysis of this novel model is presented. In fact, the morphing sequence is no longer retrieved in a post-processing step. Instead, the color values are part of the feature vector. Different from the prior proceedings article, we prove the existence of time discrete geodesics in feature space, present a time continuous model and discuss the issue of convergence of the discrete functionals.

Notation Throughout this paper, we assume that the image domain $\Omega \subset \mathbb {R}^n$ for $n\in \{2,3\}$ is bounded and strongly Lipschitz. We use standard notation for Lebesgue and Sobolev spaces from the image domain $\Omega$ to a Banach space *X*, i.e., $L^p(\Omega ,X)$ and $H^m(\Omega ,X)$ and omit *X* if the space is clear from the context. The associated norms are denoted by $\Vert \cdot \Vert _{L^p(\Omega )}$ and $\Vert \cdot \Vert _{H^m(\Omega )}$, respectively, and the seminorm in $H^m(\Omega )$ is given by $|\cdot |_{H^m(\Omega )}$, i.e.,

$$\begin{aligned} |f|_{H^m(\Omega )}=\Vert D^m f\Vert _{L^2(\Omega )}\,,\quad \Vert f\Vert _{H^m(\Omega )}^2=\sum _{j=0}^m|f|_{H^j(\Omega )}^2 \end{aligned}$$

for $f\in H^m(\Omega )$. We use the notation $C^{k,\alpha }(\overline{\Omega },X)$ for Hölder spaces of order $k\ge 0$ with regularity $\alpha \in (0,1]$, the corresponding (semi)norm is

$$\begin{aligned} |f|_{C^{0,\alpha }({\overline{\Omega }})}=&\sup _{x\ne y\in \Omega }\frac{|f(x)-f(y)|}{|x-y|^\alpha }\,,\\ \Vert f\Vert _{C^{k,\alpha }({\overline{\Omega }})}=&\Vert f\Vert _{C^k({\overline{\Omega }})}+\sum _{|\beta |=k}|D^{\beta } f|_{C^{0,\alpha }(\overline{\Omega })}\,. \end{aligned}$$

The symmetric part of a matrix $A\in \mathbb {R}^{n,n}$ is denoted by $A^\mathrm {sym}$, i.e., $A^\mathrm {sym}=\frac{1}{2}(A+A^\top )$ and the symmetrized Jacobian of a differentiable function $\phi$ by $\varepsilon [\phi ]=(D\phi )^{\mathrm {sym}}$. We denote by ${\mathrm {GL}}^+(n)$ the elements of ${\mathrm {GL}}(n)$ with positive determinant, and by ${\mathbb {1}}$ both the identity map and the identity matrix. Finally, $\dot{f}$ refers to the temporal derivative of a differentiable function *f*.

Organization This paper is structured as follows: in Sect. 2, we review the classical metamorphosis model and present its extension to deep feature spaces. Then, in Sect. 3 we introduce the time discrete deep feature metamorphosis model and prove the existence of geodesic paths. In Sect. 4, we present a time continuous metamorphosis model and comment on the Mosco convergence in deep feature space. The fully discrete model and the optimization scheme using the iPALM algorithm are presented in Sect. 5. Finally, in Sect. 6 several examples demonstrate the applicability of the proposed methods to real image data.

## Metamorphosis Model

In this section, we briefly review the classical flow of diffeomorphism model and the metamorphosis model as its generalization. Then, we extend the metamorphosis model to the space of deep features, where we additionally incorporate an anisotropic regularization.

### Flow of Diffeomorphism

In what follows, we present a very short exposition of the flow of diffeomorphism model and refer the reader to [3, 10, 14, 18] for further details. In the flow of diffeomorphism model, the temporal change of image intensities is determined by a *family of diffeomorphisms* $(\psi (t))_{t\in [0,1]}:{\overline{\Omega }}\rightarrow \mathbb {R}^n$ describing a flow transporting image intensities along particle paths. The main assumption of this model is the *brightness constancy assumption*, which is equivalent to a vanishing material derivative $\frac{D}{\partial t}u={\dot{u}}+v\cdot Du$ along a path $(u(t))_{t\in [0,1]}$ in the space of images, where $v(t)={\dot{\psi }}(t)\circ \psi ^{-1}(t)$ denotes the time-dependent *Eulerian velocity*. The Riemannian space of images is endowed with the following metric and path energy

$$\begin{aligned} g_{\psi _t}(\dot{\psi }_t,\dot{\psi }_t)&=\int _\Omega L[v,v]\,\mathrm {d}x\,,\\ \mathcal {E}_{\psi _t}[(\psi _t)_{t\in [0,1]}]&=\int ^1_0 g_{\psi _t}(\dot{\psi }_t,\dot{\psi }_t)\,\mathrm {d}t\,. \end{aligned}$$

Note that we use $\psi _t$ as a shortcut for the function $x\mapsto \psi (t,x)$. Here, the quadratic form *L* is the higher order elliptic operator

$$\begin{aligned} L[v,v]= \frac{\lambda }{2} (\mathrm {tr}\varepsilon [v])^2 + \mu \mathrm {tr}(\varepsilon [v]^2) + \gamma |D^m v|^2, \end{aligned}$$

where $m>1+\frac{n}{2}$ and $\lambda ,\mu ,\gamma >0$. Physically, the metric $g_{\psi _t}({\dot{\psi }}_t,{\dot{\psi }}_t)$ describes the viscous dissipation in a multipolar fluid model as investigated by Nečas and Šilhavý [21]. The first two terms of the integrand represent the dissipation density in a Newtonian fluid and the third term can be regarded as a higher order measure for friction. Following [10, Theorem 2.5], paths with a finite energy, which connect two diffeomorphisms $\psi _0=\psi _A$ and $\psi _1=\psi _B$, are actually one-parameter families of diffeomorphisms. Given two image intensity functions $u_A,u_B\in L^2(\Omega )$, an associated geodesic path is a family of images $u=(u(t):\Omega \rightarrow \mathbb {R})_{t\in [0,1]}$ with $u(0,\cdot )=u_A(\cdot )$ and $u(1,\cdot )=u_B(\cdot )$, which minimizes the path energy. The resulting flow of images is given by $u(t,\cdot )=u_A\circ \psi ^{-1}_t(\cdot )$.

### Metamorphosis Model in Image Space

The metamorphosis approach originally proposed by Miller et al. in [16, 29, 30] generalizes the flow of diffeomorphism model by allowing for image intensity variations along motion paths and penalizing the squared material derivative in the metric. Under the assumption that the image path $u$ is sufficiently smooth, the metric and the path energy read as

$$\begin{aligned} g({\dot{u}},{\dot{u}})&=\min _{v:{\overline{\Omega }}\rightarrow \mathbb {R}^n}\int _\Omega L[v,v]+\frac{1}{\delta }z^2\,\mathrm {d}x\,,\\ \mathcal {E}[u]&=\int _0^1 g({\dot{u}}(t),{\dot{u}}(t))\,\mathrm {d}t \end{aligned}$$

for a penalization parameter $\delta >0$, where $z=\frac{D}{\partial t}u={\dot{u}}+v\cdot Du$ denotes the material derivative of $u$. The Lagrangian formulation of this variation of the image intensity along motion trajectories can be phrased as follows: for all $s,t\in [0,1]$ we have

1 $$\begin{aligned} u(t,\psi _t)-u(s,\psi _s)=\int _s^t z(r,\psi _r)\,\mathrm {d}r\,. \end{aligned}$$

Hence, the flow of diffeomorphism model is the limit case of the metamorphosis model for $\delta \rightarrow 0$. This definition of the metric has two major drawbacks: In general, paths in the space of images do not exhibit any smoothness properties (neither in space nor time), and therefore the evaluation of the material derivative is not well-defined. Moreover, since different pairs $(v,\frac{D}{\partial t}u)$ of velocity fields and material derivatives can imply the same time derivative of the image path ${\dot{u}}$, the restriction to equivalence classes of pairs is required, where two pairs are equivalent if and only if they induce the same temporal change of the image path ${\dot{u}}$.

To tackle both problems, Trouvé and Younes [29] proposed a nonlinear geometric structure in the space of RGB images $\mathcal {I}:=L^2(\Omega ,\mathbb {R}^3)$. In detail, for a given image path $u\in L^2([0,1],\mathcal {I})$ and an associated velocity field $v\in L^2((0,1),\mathcal {V})$, where $\mathcal {V}:=H^{m}(\Omega ,\mathbb {R}^n)\cap H^{1}_0(\Omega ,\mathbb {R}^n)$ denotes the velocity space, the *weak material derivative* $z\in L^2((0,1),L^2(\Omega ,\mathbb {R}^3))$ is incorporated in the model, which is implicitly given by

2 $$\begin{aligned} \int _0^1\int _\Omega \eta z\,\mathrm {d}x\,\mathrm {d}t=-\int _0^1\int _\Omega (\partial _t\eta +{\mathrm {div}}(v\eta ))u\,\mathrm {d}x\,\mathrm {d}t \end{aligned}$$

for a smooth test function $\eta \in C^{\infty }_c((0,1)\times \Omega )$. We consider (*v*, *z*) as a tangent vector in the tangent space of $\mathcal {I}$ at the image $u$ and write $(v,z) \in T_u\mathcal {I}$ defined by (2). Indeed, (*v*, *z*) represents a variation of the image $u$ via transport and change of intensity. This (weak) formulation and the consideration of equivalence classes of motion fields and material derivatives inducing the same temporal change of the image intensity gives rise to the notion $H^1([0,1],\mathcal {I})$ for regular paths in the space of images. For details, we refer the reader to [29]. The *path energy in the metamorphosis model* for a regular path $u\in H^1([0,1],\mathcal {I})$ is then defined as

3 $$\begin{aligned} \mathcal {E}[u]=\int _0^1\inf _{(v,z) \in T_u\mathcal {I}} \int _\Omega L[v,v]+\frac{1}{\delta }z^2\,\mathrm {d}x\,\mathrm {d}t\,. \end{aligned}$$

Then, image morphing of two input images $u_A,u_B\in \mathcal {I}$ amounts to computing a shortest geodesic path $u\in H^1([0,1],\mathcal {I})$ in the metamorphosis model, which is defined as a minimizer of the path energy in the class of regular curves such that $u(0)=u_A$ and $u(1)=u_B$. The existence of a shortest geodesic is proven in [29, Theorem 6]. Note that the infimum in (3) is attained, which is shown in [29, Proposition 1 & Theorem 2].

### Metamorphosis Model in Deep Feature Space

In this subsection, we extend the metamorphosis model to images as maps into a deep feature space with the aim to increase the reliability and robustness of the resulting morphing. To further improve the quality of the deformations, we incorporate an anisotropic regularization of the deformation field. We will compute geodesic paths in the *feature space* $\mathcal {F}:=L^2(\Omega ,\mathbb {R}^{3+C}$) for $C\ge 0$. Here, the first part $u\in \mathcal {I}$ of a feature vector $f=(u,{\tilde{f}})\in \mathcal {F}$ encodes the RGB image intensity values, the remaining component ${\tilde{f}}\in L^2(\Omega ,\mathbb {R}^C)$ represents deep features, which are high-dimensional local image patterns describing the local structure of the image as a superposition on different levels of a multiscale image approximation. Let us denote by $\mathcal {P}$ the projection onto the image component of a feature, i.e., $\mathcal {P}[f]=u$. To compute the geodesic sequence in the deep feature space, we extract the features  $\mathbf {F}(u_A),\mathbf {F}(u_B) \in L^2(\Omega ,\mathbb {R}^C)$ from the fixed input images $u_A,u_B\in \mathcal {I}$ and define for a fixed (small) $\eta >0$

$$\begin{aligned} f_A=(\eta u_A,\mathbf {F}(u_A))\,,\quad f_B=(\eta u_B,\mathbf {F}(u_B))\,. \end{aligned}$$

The computation of the VGG features is composed of convolution operators and nonlinear ReLU activation functions which are both continuous mappings. Hence, it is reasonable to assume in our mathematical model that the mapping $\mathbf {F}:\mathcal {I}\rightarrow L^2(\Omega ,\mathbb {R}^C)$ is *continuous*. Following [28], we define for the fully discrete model discussed in Sect. 5 a discrete feature operator to incorporate semantic information in image morphing based on convolutional neural networks, where *C* ranges from 64 to 512. The parameter $\eta$ is used to scale down the RGB component mainly needed to compute the anisotropy (see below) and to primarily focus on the actual VGG features when estimating the transport.

Next, we include an anisotropic elliptic operator *L* in our model to properly account for image structures such as sharp edges or corners. To this end, we consider an *anisotropy operator* $a:\mathcal {I}\rightarrow L^\infty (\Omega )$ fulfilling the following assumptions:

1. *boundedness and coercivity*: $c_a<a[u](x)<C_a$ for $0< c_a<C_a$ and all $u\in \mathcal {I}$ and a.e. $x\in \Omega$,
2. *compactness*: $u_k\rightharpoonup u$ in $\mathcal {I}$ implies $a[u_k]\rightarrow a[u]$ in $L^\infty (\Omega )$,
3. *Lipschitz continuity*: for all neighborhoods $\mathcal {U}\subset \mathcal {I}$ there exists $L_a>0$ such that $\Vert a[u]-a[{\tilde{u}}]\Vert _{L^\infty }\le L_a\Vert u-{\tilde{u}}\Vert _{\mathcal {I}}$ for all $u,{\tilde{u}}\in \mathcal {U}$.

In the numerical experiments, we use the operator [23]

4 $$\begin{aligned} a[u](x)=\exp \left( -\frac{\Vert (\mathcal {G}_{\rho }*D\mathcal {G}_{\sigma }*u)(x)\Vert _2^2}{\xi _1}\right) +\xi _2, \end{aligned}$$

for fixed $\xi _1,\xi _2>0$, where $\mathcal {G}_{\sigma }$, $\mathcal {G}_{\rho }$ are the Gaussian kernels with standard deviation $\sigma ,\rho >0$. Note that (4) satisfies (A1)–(A3). In fact, the anisotropy operator *a* is a scale factor for the elliptic operator of the deformation field, which nearly vanishes in the proximity of interfacial structures. Thus, large deformation gradients are less penalized in these regions and consequently sharp edges can be better preserved along geodesic paths. Now we are in the position to introduce the variational model for deep feature metamorphosis. Instead of generalizing the definition of regular paths and adapting the notion of a weak material derivative (2) originally proposed by Trouvé and Younes, we follow the relaxed material derivative approach proposed in [13], in which the material derivative quantity is retrieved from a variational inequality. In [13, Section 3], the equivalence of this energy functional and (3) in the isotropic case has been shown. Let $\psi$ as above denote the Lagrangian flow map induced by the Eulerian motion field with ${\dot{\psi }}_t(x)=v(t,\psi _t(x))$ and $\psi _0(x)=x$. Then, we replace the equality (1) (rephrased for the feature map $f$ as $f(t,\psi _t)-f(s,\psi _s)=\int _s^t \tilde{z}(r,\psi _r) \,\mathrm {d}r$ with ${\tilde{z}}\in L^2((0,1)\times \Omega ,\mathbb {R}^{3+C})$ being the weak material derivative) by the inequality

5 $$\begin{aligned} |f(t,\psi _t(x))-f(s,\psi _s(x))|\le \int _s^t z(r,\psi _r(x))\,\mathrm {d}r \end{aligned}$$

for a.e. $x\in \Omega$ and all $1\ge t > s \ge 0$, where formally the scalar valued $z=|{\tilde{z}}|$ replaces the actually vector-valued material derivative. In fact, this inequality defines a set $\mathcal {C}(f)$ of admissible pairs (*v*, *z*) given a path $f$ in $L^2([0,1],\mathcal {F})$. This relaxed approach will turn out to be very natural when it comes to lower semicontinuity of the path energy in the context of the existence proof for geodesic paths. For more details, we refer the reader to Sect. 4.

#### Definition 1

(*Continuous path energy*) We consider the anisotropic elliptic operator

$$\begin{aligned} L[{\tilde{a}},v,v]={\tilde{a}} \left( \frac{\lambda }{2}(\mathrm {tr}\varepsilon [v])^2+\mu \mathrm {tr}(\varepsilon [v]^2)\right) +\gamma |D^m v|^2 \end{aligned}$$

for an anisotropy operator ${\tilde{a}}\in L^\infty (\Omega )$, a velocity field $v\in \mathcal {V}$ and $\gamma ,\mu ,\lambda >0$. Then, we define the *path energy*

6 $$\begin{aligned} \mathcal {E}[f]=\int _0^1\inf _{(v,z)\in \mathcal {C}(f)}\int _\Omega L[a[\mathcal {P}[f]],v,v]+\frac{1}{\delta }z^2\,\mathrm {d}x\,\mathrm {d}t \end{aligned}$$

for a path $f\in L^2([0,1],\mathcal {F})$, where

$$\begin{aligned} \mathcal {C}(f)\subset L^2((0,1),\mathcal {V})\times L^2((0,1) \times \Omega ) \end{aligned}$$

denotes the set of admissible pairs of the velocity and a scalar quantity *z* fulfilling (5).

Let us stress that the anisotropy ${\tilde{a}}=a[\mathcal {P}[f]]$ solely takes into account local RGB values and not the actual VGG features with their discriminative multiscale characteristics.

Geodesic curves $f\in L^2([0,1],\mathcal {F})$ in the deep feature space joining $f_A,f_B\in \mathcal {F}$ are defined as minimizers of the path energy $\mathcal {E}$ among all curves with the fixed boundary conditions $f(0)=f_A$ and $f(1)=f_B$.

#### Remark 1

One observes that a path $f\in L^2([0,1],\mathcal {F})$ in feature space with finite energy $\mathcal {E}[f]<\infty$ exhibits additional smoothness properties. Indeed, the boundedness of *v* in $L^2((0,1),H^m(\Omega ,\mathbb {R}^n))$ implies that the flow is in $\psi \in H^{1}((0,1),H^m(\Omega ,\Omega ))$ and, by using Sobolev embedding arguments, in $C^{0,\frac{1}{2}}([0,1],C^{1,\alpha }({\overline{\Omega }},{\overline{\Omega }}))$ with $\alpha \in (0,\min \{1,m-1-\frac{n}{2}\})$. The same observation holds for $\psi ^{-1}$ by noting that $\psi ^{-1}_t(\cdot )$ is the flow associated with the backward motion field $-v(1-t,\cdot )$. This together with the variational inequality (5) and *z* in $L^2((0,1)\times \Omega )$ ensures that $t \mapsto f(t,\psi (t,\cdot ))\in H^1((0,1),\mathcal {F})\subset C^{0,\frac{1}{2}}([0,1],\mathcal {F})$. Using approximation by smooth functions, one shows that $t\mapsto f(t,\cdot )\in C^{0}([0,1],\mathcal {F})$ is uniformly continuous, and by using (A3) the mapping $t\mapsto a[\mathcal {P}[f(t,\cdot )]]$ is well-defined and in $C^0([0,1],L^\infty (\Omega ))$.

## Variational Time Discretization

In this section, we develop a variational time discretization of the deep feature space metamorphosis model taking into account the approach presented in [4, 27].

We define the *time discrete pairwise energy* for two feature maps $f,{\tilde{f}}\in \mathcal {F}$ by

$$\begin{aligned} \mathcal {W}[f,{\tilde{f}}]=\min _{\phi \in \mathcal {A}} \mathcal {W}^D[a[\mathcal {P}[{\tilde{f}}]], f,{\tilde{f}},\phi ]\,, \end{aligned}$$

where $\mathcal {W}^D:L^\infty (\Omega )\times \mathcal {F}\times \mathcal {F}\times \mathcal {A}\rightarrow \mathbb {R}$ is given by

7 $$\begin{aligned}&\mathcal {W}^D[{\tilde{a}},f,\tilde{f},\phi ]\nonumber \\&\quad = \int _\Omega {\tilde{a}}\mathrm {W}(D\phi ) +\gamma |D^m \phi |^2+\frac{1}{\delta }|\tilde{f}\circ \phi -f|^2\,\mathrm {d}x\,. \end{aligned}$$

Here, the *set of admissible deformations* is

$$\begin{aligned} \mathcal {A}=\{\phi \in H^m(\Omega ,\Omega ):&\det (D\phi )>0\text { a.e. in }\Omega ,\\&\phi |_{\partial \Omega }={\mathbb {1}}\}. \end{aligned}$$

Note that the anisotropy operator only depends on the image component of the second feature ${\tilde{f}}$ in the pairwise energy. We make the following assumptions with respect to the *energy density function* $\mathrm {W}$:

1. $\mathrm {W}:\mathbb {R}^{n,n}\rightarrow \mathbb {R}^+_0$ and $\mathrm {W}\in C^4({\mathrm {GL}}^+(n))$ is polyconvex and $\mathrm {W}({\mathbb {1}})=0$, $D\mathrm {W}({\mathbb {1}})=0$,
2. there exist constants $C_{\mathrm {W},1},C_{\mathrm {W},2},r_\mathrm {W}>0$ such that for all $A\in {\mathrm {GL}}^+(n)$ the growth estimates
  $$\begin{aligned} \mathrm {W}(A)&\ge C_{\mathrm {W},1}\vert A^\mathrm {sym}-{\mathbb {1}}\vert ^2\,,&\text {if }\vert A-{\mathbb {1}}\vert <r_\mathrm {W}\,,\\ \mathrm {W}(A)&\ge C_{\mathrm {W},2}\,,&\text {if }\vert A-{\mathbb {1}}\vert \ge r_\mathrm {W}\end{aligned}$$
  hold true,
3. for all $A\in \mathbb {R}^{n,n}$ the relation
  $$\begin{aligned} \frac{1}{2}D^2\mathrm {W}({\mathbb {1}})(A,A)=\frac{\lambda }{2}(\mathrm {tr}A)^2+\mu \mathrm {tr}((A^\mathrm {sym})^2) \end{aligned}$$
  holds true.

The first two assumptions ensure existence of a minimizing deformation in (7), and the third is a consistency assumption with respect to the differential operator *L* required to guarantee that the below defined discrete path energy is consistent with the time continuous path energy (6).

The particular energy density function

8 $$\begin{aligned} \mathrm {W}(D\phi )=\frac{\lambda }{2}\left( e^{(\log \det (D\phi ))^2}-1\right) +\mu |\varepsilon [\phi ]-{\mathbb {1}}|^2 \end{aligned}$$

used for all numerical experiments satisfies (W1)–(W3). The first term enforces the positivity of the determinant of the Jacobian matrix of a deformation and favors a balance of shrinkage and growth as advocated in [5, 9], while the second term penalizes large deviations of the deformation from the identity. Here, the positivity constraint of the determinant of the Jacobian of the deformations prohibits interpenetration of matter [2].

We proceed with the definition of the discrete path energy and the discrete geodesic between two features $f_A=(\eta u_A,\mathbf {F}(u_A)), f_B=(\eta u_B,\mathbf {F}(u_B))\in \mathcal {F}$.

### Definition 2

(*Discrete path energy*) Let $K\ge 1$ and $f_0=f_A,f_K=f_B\in \mathcal {F}$. The *discrete path energy* $\mathbf {E}^K$ for a discrete $(K+1)$-path $\mathbf {f}=(f_0,\dots ,f_K)\in \mathcal {F}^{K+1}$ is defined as

9 $$\begin{aligned} {\mathbf {E}^K}[\mathbf {f}]:=K\sum _{k=1}^K\mathcal {W}[f_{k-1},f_k]\,. \end{aligned}$$

A *discrete geodesic path* morphing $f_A \in \mathcal {F}$ into $f_B \in \mathcal {F}$ is a discrete $(K+1)$-tuple that minimizes $\mathbf {E}^K$ over all discrete paths $\mathbf {f}=(f_A,{{{\hat{\mathbf{f}}}}},f_B)\in \mathcal {F}^{K+1}$ with ${{{\hat{\mathbf{f}}}}}=(f_1,\ldots ,f_{K-1})\in \mathcal {F}^{K-1}$.

For arbitrary vectors $\mathbf {f}=(f_0,\ldots ,f_K)\in \mathcal {F}^{K+1}$ and $\varvec{\Phi }=(\phi _1,\ldots ,\phi _K)\in \mathcal {A}^K$ we set

10 $$\begin{aligned} {\mathbf {E}^{K,D}}[\mathbf {f},\varvec{\Phi }]:=K\sum _{k=1}^{K}\mathcal {W}^D[a[\mathcal {P}[f_k]], f_{k-1},f_k,\phi _k]\,. \end{aligned}$$

In what follows, we will investigate the existence of discrete geodesic curves in the time discrete deep feature space metamorphosis model. To this end, we combine the proofs of the local well-posedness of the pairwise energy $\mathcal {W}$ with the existence result of a feature vector minimizing $\mathbf {E}^{K,D}$ for a fixed vector of deformations. We remark that the structure of all proofs is similar to the corresponding proofs in [4, 11] and we focus on the adaptations necessitated by the anisotropic regularization.

The following lemma, which provides an estimate for the $H^m(\Omega )$-norm of the displacement, is crucial for the well-posedness of the energy.

### Lemma 1

Let (W1)–(W2) and (A1) be satisfied. Then, there exists a continuous and monotonically increasing function $\theta :\mathbb {R}^+_0\rightarrow \mathbb {R}^+_0$ with $\theta (0)=0$, which only depends on $\Omega$, *m*, *n*, $\gamma$, $c_a$, $C_{\mathrm {W},1}$, $C_{\mathrm {W},2}$ and $r_{\mathrm {W}}$, such that

$$\begin{aligned} \Vert \phi -{\mathbb {1}}\Vert _{H^m(\Omega )}\le \theta \left( \mathcal {W}^D[a[\mathcal {P}[{\tilde{f}}]], f,{\tilde{f}},\phi ]\right) \end{aligned}$$

for all $f, {\tilde{f}} \in \mathcal {F}$ and all $\phi \in \mathcal {A}$. Furthermore, $\theta (x)\le C(x+x^2)^{\frac{1}{2}}$ for a constant $C>0$.

### Proof

Set ${\overline{\mathcal {W}}}=\mathcal {W}^D[a[\mathcal {P}[{\tilde{f}}]], f, {\tilde{f}}, \phi ]$. An application of the Gagliardo–Nirenberg inequality [22] yields

11 $$\begin{aligned} \Vert \phi -{\mathbb {1}}\Vert _{H^m(\Omega )}\le C(\Vert \phi -{\mathbb {1}}\Vert _{L^2(\Omega )}+|\phi -{\mathbb {1}}|_{H^m(\Omega )})\,. \end{aligned}$$

The last term in (11) is bounded by

12 $$\begin{aligned} |\phi -{\mathbb {1}}|_{H^m(\Omega )}=|\phi |_{H^m(\Omega )} \le \sqrt{\frac{\overline{\mathcal {W}}}{\gamma }}\,. \end{aligned}$$

By using the embedding of $H^m(\Omega ,\Omega )$ into $C^{1,\alpha }({\overline{\Omega }},{\overline{\Omega }})$ and the uniform boundedness of the minimizing sequence in $L^2(\Omega ,\Omega )$ we get $\Vert \phi -{\mathbb {1}}\Vert _{C^{1,\alpha }({\overline{\Omega }})} \le C+C\sqrt{\overline{\mathcal {W}}}\,.$ To control the lower order term appearing on the right-hand side of (11), we define $\mathcal {S}=\{x\in \Omega :|D\phi (x)-{\mathbb {1}}|<r_\mathrm {W}\}$ and use (A1) and (W2) to obtain

$$\begin{aligned} |\Omega \backslash \mathcal {S}|c_aC_{\mathrm {W},2}\le \int _\Omega a[\mathcal {P}[{\tilde{f}}]]\mathrm {W}(D\phi ) \,\mathrm {d}x\le \overline{\mathcal {W}}\,, \end{aligned}$$

which implies $|\Omega \backslash \mathcal {S}|\le \frac{\overline{\mathcal {W}}}{c_aC_{\mathrm {W},2}}$. Hence, by the embedding $H^m(\Omega ,\Omega )\hookrightarrow C^1({\overline{\Omega }},{\overline{\Omega }})$ we infer

13 $$\begin{aligned}&\int _\Omega |\varepsilon [\phi ]-{\mathbb {1}}|^2 \,\mathrm {d}x\nonumber \\&\quad \le \int _\mathcal {S}\frac{\mathrm {W}(D\phi )}{C_{\mathrm {W},1}} \,\mathrm {d}x +|\Omega \backslash \mathcal {S}|\left( C+C\sqrt{\overline{\mathcal {W}}}\right) ^2\nonumber \\&\quad \le \frac{\overline{\mathcal {W}}}{C_{\mathrm {W},1}}+ \frac{\overline{\mathcal {W}}}{c_aC_{\mathrm {W},2}}\left( C+C\overline{\mathcal {W}}\right) \,. \end{aligned}$$

We remark that the inequality

14 $$\begin{aligned} \Vert \phi -{\mathbb {1}}\Vert _{L^2(\Omega )}\le C\Vert \varepsilon [\phi ]-{\mathbb {1}}\Vert _{L^2(\Omega )} \end{aligned}$$

holds true, which follows from Korn’s inequality and the Poincaré inequality. Thus, the lemma follows by combining (11), (12), (13) and (14). $\square$

### Proposition 1

(Well-posedness of $\mathcal {W})$ Let $f\in \mathcal {F}$ be a fixed feature vector. Under the assumptions (W1)–(W2) and (A1), there exists a constant $C_\mathcal {W}$ (depending on $\Omega ,m,n,\gamma ,\delta ,\mu ,\lambda ,c_a,C_{\mathrm {W},1},C_{\mathrm {W},2},r_{\mathrm {W}}$) such that for every fixed

15 $$\begin{aligned} {\tilde{f}}\in \left\{ g\in \mathcal {F}:\Vert f-g\Vert _{\mathcal {F}}<C_\mathcal {W}\right\} \end{aligned}$$

there exists $\phi \in \mathcal {A}$ which minimizes $\mathcal {W}^D[a[\mathcal {P}[{\tilde{f}}]],f,{\tilde{f}},\cdot ]$ defined in (7) and $\phi$ is a $C^1(\Omega ,\Omega )$-diffeomorphism.

### Proof

For fixed $f\in \mathcal {F}$, let ${\tilde{f}}$ be a feature vector satisfying (15) for a constant $C_\mathcal {W}$ specified below. Let $\{\phi ^j\}_{j\in \mathbb {N}}\in \mathcal {A}$ be any sequence such that the mismatch $\mathcal {W}^D[a[\mathcal {P}[{\tilde{f}}]], f,{\tilde{f}},\phi ^j]$ converges to

$$\begin{aligned} \underline{\mathbf {W}}=\inf _{\phi \in \mathcal {A}}\mathcal {W}^D [a[\mathcal {P}[{\tilde{f}}]], f,{\tilde{f}},\phi ]\ge 0. \end{aligned}$$

Since ${\mathbb {1}}\in \mathcal {A}$, we can deduce using (W1) that

$$\begin{aligned}&\underline{\mathbf {W}}\le \mathcal {W}^D[a[\mathcal {P}[{\tilde{f}}]], f,{\tilde{f}},\phi ^j]\\&\quad \le \overline{\mathbf {W}}:=\mathcal {W}^D [a[\mathcal {P}[{\tilde{f}}]],f,{\tilde{f}},{\mathbb {1}}] =\frac{1}{\delta }\Vert {\tilde{f}}-f\Vert _{\mathcal {F}}^2 <\frac{C_\mathcal {W}^2}{\delta } \end{aligned}$$

for all $j\in \mathbb {N}$. Using again the Gagliardo–Nirenberg inequality we infer that $\{\phi ^j\}_{j\in \mathbb {N}}$ is uniformly bounded in $H^m(\Omega ,\Omega )$ because of the estimate $|\phi ^j|_{H^m(\Omega )}^2\le \frac{\overline{\mathbf {W}}}{\gamma }$. Due to the reflexivity of $H^m(\Omega ,\Omega )$, there exists a weakly convergent subsequence (not relabeled) such that $\phi ^j\rightharpoonup \phi$ in $H^m(\Omega ,\Omega )$. By using the Sobolev embedding theorem as well as the Arzelà–Ascoli theorem, we can additionally infer that for a subsequence (again not relabeled) $\phi ^j\rightarrow \phi$ in $C^{1,\alpha }({\overline{\Omega }},{\overline{\Omega }})$ for $\alpha \in (0,m-1-\frac{n}{2})$ holds true. Then, Lemma 1 implies

$$\begin{aligned} \Vert \phi ^j-{\mathbb {1}}\Vert _{C^1({\overline{\Omega }})}\le C\theta (\overline{\mathbf {W}})<C\theta (\delta ^{-1}C_{\mathcal {W}}^2)\,. \end{aligned}$$

Thus, by choosing $C_\mathcal {W}$ sufficiently small and taking into account the Lipschitz continuity of the determinant we obtain $\Vert \det (D\phi ^j)-1\Vert _{L^\infty (\Omega )}\le C_{\det }$ for a constant $C_{\det }\in (0,1)$ and all $j\in \mathbb {N}$, which implies $\det (D\phi ^j)\ge C>0$ for a constant *C*. Note that all estimates remain valid for the limit deformation $\phi \in \mathcal {A}$. By [7, Theorem 5.5-2] the deformations $\{\phi ^j\}_{j\in \mathbb {N}}$ and $\phi$ are $C^1(\Omega ,\Omega )$-diffeomorphisms. Finally, (W1) and the lower semicontinuity of the seminorm imply

$$\begin{aligned}&\liminf _{j\rightarrow \infty }\int _\Omega a[\mathcal {P}[{\tilde{f}}]] \mathrm {W}(D\phi ^j)+\gamma |D^m\phi ^j|^2\,\mathrm {d}x\\&\quad \ge \int _\Omega a[\mathcal {P}[{\tilde{f}}]] \mathrm {W}(D\phi )+\gamma |D^m\phi |^2\,\mathrm {d}x\,. \end{aligned}$$

It remains to verify that

16 $$\begin{aligned} \Vert {\tilde{f}}\circ \phi ^j-f\Vert _{\mathcal {F}}\rightarrow \Vert {\tilde{f}}\circ \phi -f\Vert _{\mathcal {F}} \end{aligned}$$

as $j\rightarrow \infty$. To this end, we approximate ${\tilde{f}}$ by smooth functions ${\tilde{f}}^i\in C^\infty (\Omega ,\mathbb {R}^{3+C})$ with $\Vert {\tilde{f}}-{\tilde{f}}^i\Vert _{\mathcal {F}}\rightarrow 0$. Then, using the transformation formula we obtain

$$\begin{aligned}&\Vert {\tilde{f}}\circ \phi ^j-{\tilde{f}} \circ \phi \Vert _{\mathcal {F}}\\&\quad \le \Vert {\tilde{f}}\circ \phi ^j-{\tilde{f}}^i \circ \phi ^j\Vert _{\mathcal {F}}+\Vert {\tilde{f}}^i \circ \phi ^j-{\tilde{f}}^i\circ \phi \Vert _{\mathcal {F}}\\&\qquad +\Vert {\tilde{f}}^i\circ \phi -{\tilde{f}} \circ \phi \Vert _{\mathcal {F}}\\&\quad \le \Vert {\tilde{f}}-{\tilde{f}}^i\Vert _{\mathcal {F}} \Big (\Vert \det (D(\phi ^j)^{-1})\Vert _{L^\infty (\Omega )}^{\frac{1}{2}}\\&\qquad +\Vert \det (D\phi ^{-1})\Vert _{L^\infty (\Omega )}^{\frac{1}{2}}\Big ) +\Vert D{\tilde{f}}_i\Vert _{L^\infty (\Omega )}\Vert \phi ^j -\phi \Vert _{L^2(\Omega )}\,, \end{aligned}$$

where $\det (D(\phi ^j))^{-1}$ and $\det (D(\phi ))^{-1}$ are pointwise estimated by $(1-C_{\det })^{\frac{1}{2}}\,.$ Finally, by first choosing *i* and then *j* we obtain (16) and thereby verify the claim. $\square$

This proposition guarantees the existence of an admissible vector of deformations $\varvec{\Phi }\in \mathcal {A}^K$ such that $\mathbf {E}^{K,D}[\mathbf {f},\varvec{\Phi }]=\mathbf {E}^K[\mathbf {f}]$ provided that each pair of features $(f_k,f_{k+1})$ contained in $\mathbf {f}=(f_0,\ldots ,f_K)\in \mathcal {F}^{K+1}$ satisfies (15).

In what follows, we prove the existence of an energy minimizing vector of features for a fixed vector of deformations.

### Proposition 2

Let $K\ge 2$, $f_A,f_B\in \mathcal {F}$ and $\varvec{\Phi }=(\phi _1,\ldots ,\phi _K)\in \mathcal {A}^K$ be fixed. We assume that the deformations satisfy

17 $$\begin{aligned} \min _{k\in \{1,\ldots ,K\}}\min _{x\in {\overline{\Omega }}} \det (D\phi _k(x))\ge c_{\det } \end{aligned}$$

for a constant $c_{\det }>0$. Then, under the assumptions (W1)–(W2) and (A1)–(A2) there exists a feature vector $\mathbf {f}$ with $f_0=f_A$ and $f_K=f_B$ such that

$$\begin{aligned} {\mathbf {E}^{K,D}}[\mathbf {f},\varvec{\Phi }]=\inf \left\{ {\mathbf {E}^{K,D}}[(f_A, {{{\hat{\mathbf{g}}}}},f_B),\varvec{\Phi }]:{{{\hat{\mathbf{g}}}}}\in \mathcal {F}^{K-1}\right\} \,. \end{aligned}$$

### Proof

We consider a minimizing sequence of features ${{{\hat{\mathbf{f}}}}}^j=(f^j_1,\ldots ,f^j_{K-1})\in \mathcal {F}^{K-1}$, $j\in \mathbb {N}$, for the energy ${{{\hat{\mathbf{g}}}}}\mapsto {\mathbf {E}^{K,D}}[(f_A,{{{\hat{\mathbf{g}}}}},f_B),\varvec{\Phi }]$. Then,

$$\begin{aligned} 0&\le {\mathbf {E}^{K,D}}[(f_A,{{{\hat{\mathbf{f}}}}}^j,f_B),\varvec{\Phi }]\\&\le {\mathbf {E}^{K,D}}[(f_A, (f_A,\ldots ,f_A),f_B),\varvec{\Phi }] =:{\overline{\mathbf {E}^{K,D}}}\,. \end{aligned}$$

A straightforward computation reveals

$$\begin{aligned} {\overline{\mathbf {E}^{K,D}}}&\quad \le K\sum _{k=1}^K C_a\Vert \mathrm {W}(D\phi _k)\Vert _{L^1(\Omega )}+\gamma \Vert \phi _k\Vert _{H^m(\Omega )}^2\\&+\frac{CK^2}{\delta }\left( (1+c_{\det }^{-1}) \Vert f_A\Vert _{\mathcal {F}}^2+c_{\det }^{-1} \Vert f_B\Vert _{\mathcal {F}}^2\right) \,, \end{aligned}$$

where we used (A1), (17) and the transformation formula. Furthermore, again by (17) one obtains

18 $$\begin{aligned} \Vert f_k^j\Vert _{\mathcal {F}}\le&\Vert f_{k+1}^j \circ \phi _{k+1}-f_k^j\Vert _{\mathcal {F}}+ \Vert f_{k+1}^j\circ \phi _{k+1}\Vert _{\mathcal {F}}\nonumber \\&\quad \le \sqrt{\frac{\delta {\overline{\mathbf {E}^{K,D}}}}{K}} +c_{\det }^{-\frac{1}{2}}\Vert f_{k+1}^j\Vert _{\mathcal {F}}\,. \end{aligned}$$

Thus, an induction argument (starting from $k=K-1$) shows that ${{{\hat{\mathbf{f}}}}}^j=(f^j_1,\ldots ,f^j_{K-1})$ is uniformly bounded in $\mathcal {F}^{K-1}$ independently of *j*, which implies for a subsequence (not relabeled) ${{{\hat{\mathbf{f}}}}}^j\rightharpoonup {{{\hat{\mathbf{f}}}}}$ in $\mathcal {F}^{K-1}$.

In what follows, we prove the weak lower semicontinuity of the discrete path energy along the minimizing sequence. We observe that (A2) implies $a[\mathcal {P}[f^j_k]]\rightarrow a[\mathcal {P}[f_k]]$ in $L^\infty (\Omega )$, which yields

$$\begin{aligned} \lim _{j\rightarrow \infty }\int _\Omega a[\mathcal {P}[f^j_k]] \mathrm {W}(D\phi _k)\,\mathrm {d}x=\int _\Omega a[\mathcal {P}[f_k]]\mathrm {W}(D\phi _k)\,\mathrm {d}x \end{aligned}$$

for every $k=1,\ldots ,K$. It remains to verify the weak lower semicontinuity of the matching functional, i.e.,

19 $$\begin{aligned} \Vert f_k\circ \phi _k-f_{k-1} \Vert _{\mathcal {F}}^2\le \liminf _{j\rightarrow \infty } \Vert f^j_k\circ \phi _k-f^j_{k-1}\Vert _\mathcal {F}^2 \end{aligned}$$

for every $k=1,\dots ,K$. To this end, we first show $f_k^j\circ \phi _k\rightharpoonup f_k\circ \phi _k$ in $\mathcal {F}$. For every $g \in \mathcal {F}$, the transformation formula yields

$$\begin{aligned}&\int _{\Omega }(f^j_k\circ \phi _k- f_k\circ \phi _k)\cdot g\,\mathrm {d}x\\&\quad = \int _{\Omega }(f^j_k-f_k) \cdot (g(\det (D\phi _k))^{-1})\circ \phi _k^{-1}\,\mathrm {d}x\,, \end{aligned}$$

which converges to 0 since $(g(\det (D\phi _k))^{-1})\circ \phi _k^{-1}\in \mathcal {F}$ due to (17). Hence, $f_k^j\circ \phi _k-f_{k-1}^j \rightharpoonup f_k\circ \phi _k-f_{k-1}$ in $\mathcal {F}$, which readily implies (19). Hence,

$$\begin{aligned} \liminf _{j\rightarrow \infty }{\mathbf {E}^{K,D}} [(f_A,{{{\hat{\mathbf{f}}}}}^j,f_B),\varvec{\Phi }] \ge {\mathbf {E}^{K,D}}[(f_A,{{{\hat{\mathbf{f}}}}},f_B),\varvec{\Phi }]\,, \end{aligned}$$

which proves the proposition. $\square$

We can now combine both previous propositions to prove the existence of discrete geodesics for the deep feature space metamorphosis model.

### Theorem 1

(Existence of discrete geodesics) Let the assumptions (W1)–(W2) and (A1)–(A2) be satisfied, $K\ge 2$ and $f_A\in \mathcal {F}$. Then, there exists a constant $C_{\mathbf {E}}>0$, which is independent of *K*, such that for every

20 $$\begin{aligned} f_B\in \left\{ g\in \mathcal {F}:\Vert g-f_A\Vert _\mathcal {F}<C_{\mathbf {E}}\sqrt{K}\right\} \end{aligned}$$

there exists ${{{\hat{\mathbf{f}}}}}\in \mathcal {F}^{K-1}$ such that

$$\begin{aligned} {\mathbf {E}^K}[(f_A,{{{\hat{\mathbf{f}}}}},f_B)] =\inf _{{{{\hat{\mathbf{g}}}}}\in \mathcal {F}^{K-1}}{\mathbf {E}^K} [(f_A,{{{\hat{\mathbf{g}}}}},f_B)] \end{aligned}$$

and the associated vector of minimizing deformations consists of $C^1(\Omega ,\Omega )$-diffeomorphisms.

### Proof

For a fixed $f_A\in \mathcal {F}$ let $f_B$ satisfy (20) for a constant $C_{\mathbf {E}}$ specified below. For $k=0,\ldots ,K$ let ${\overline{f}}_k=\frac{k}{K}f_B +(1-\frac{k}{K})f_A\in \mathcal {F}$ be a convex combination of the input features. We first note that

$$\begin{aligned} {\overline{\mathbf {E}^K}}:=&{\mathbf {E}^{K,D}} [({\overline{f}}_0,{\overline{f}}_1,\ldots ,{\overline{f}}_K), ({\mathbb {1}},\ldots ,{\mathbb {1}})]\\&\quad = \frac{K}{\delta }\sum _{k=1}^K \Vert f_{k}-f_{k-1}\Vert ^2_{\mathcal {F}}= \frac{1}{\delta }\Vert f_B-f_A\Vert _{\mathcal {F}}^2 <\frac{C_{\mathbf {E}}^2 K}{\delta } \end{aligned}$$

is a finite upper bound for the energy. Consider the minimizing sequence

$$\begin{aligned} (\mathbf {f}^j,\varvec{\Phi }^j)= ((f_0^j,\ldots ,f_K^j),(\phi _1^j,\ldots , \phi _K^j))\in \mathcal {F}^{K+1}\times \mathcal {A}^K \end{aligned}$$

for $j\in \mathbb {N}$ with $f_0^j=f_A$ and $f_K^j=f_B$ associated with the variational problem $(\mathbf {f},\varvec{\Phi })\mapsto {\mathbf {E}^{K,D}}[\mathbf {f},\varvec{\Phi }]$, which has the finite upper bound $\overline{\mathbf {E}^K}$. Following the same line of arguments as in Proposition 1, we obtain the boundedness of $\varvec{\Phi }^j$ in $H^m(\Omega ,\Omega )$, which results in a weakly convergent subsequence (not relabeled) $\varvec{\Phi }^j\rightharpoonup \varvec{\Phi }$ in $H^m(\Omega ,\Omega )$. Due to $H^m(\Omega ,\Omega )\hookrightarrow C^1({\overline{\Omega }},{\overline{\Omega }})$ one obtains $\varvec{\Phi }^j\rightarrow \varvec{\Phi }$ in $C^1({\overline{\Omega }},{\overline{\Omega }})$ for a further subsequence (not relabeled). By taking into account Lemma 1, we get

$$\begin{aligned} \Vert \phi ^j_k-{\mathbb {1}}\Vert _{C^1({\overline{\Omega }})}&\le C\Vert \phi ^j_k-{\mathbb {1}}\Vert _{H^m(\Omega )}\\&\le C\theta (K^{-1}{\overline{\mathbf {E}^K}}) \le C\theta (\delta ^{-1}C_{\mathbf {E}}^2) \end{aligned}$$

for every $j\in \mathbb {N}$ and every $k=1,\dots ,K$. By adapting $C_{\mathbf {E}}$ if necessary we can assume

$$\begin{aligned} \inf _{j\in \mathbb {N}}\min _{k=1,\ldots ,K}\min _{x\in {\overline{\Omega }}} \;\det (D\phi ^j_k(x))>c_{\det } \end{aligned}$$

for a constant $c_{\det }>0$. Taking into account [7, Theorem 5.5-2] we can conclude that $\varvec{\Phi }^j$ and $\varvec{\Phi }$ are $C^1(\Omega ,\Omega )$-diffeomorphisms. Using Proposition 2, we can replace $\mathbf {f}^j$ by the energy minimizing feature vector associated with $\varvec{\Phi }^j$, which possibly reduces the path energy. The features $\mathbf {f}^j$ are uniformly bounded in $\mathcal {F}^{K+1}$, which follows from an analogous reasoning as (18). Thus, $\mathbf {f}^j\rightharpoonup \mathbf {f}$ holds true for a subsequence (not relabeled) in $\mathcal {F}^{K+1}$, which implies $a[\mathcal {P}[f^j_k]] \rightarrow a[\mathcal {P}[f_k]]$ in $L^\infty (\Omega )$ due to (A2). Consequently, for every $k=1,\dots ,K$ we obtain

$$\begin{aligned} \liminf _{j\rightarrow \infty }\int _{\Omega }\!{a[\mathcal {P}[f^j_k]] \mathrm {W}(D\phi ^j_k)}\,\mathrm {d}x \ge \!\!\int _{\Omega }\!{a[\mathcal {P}[f_k]] \mathrm {W}(D\phi _k )}\,\mathrm {d}x\,. \end{aligned}$$

Finally, we verify the lower semicontinuity estimate

21 $$\begin{aligned} \Vert f_k\circ \phi _k-f_{k-1} \Vert _{\mathcal {F}}^2\le \liminf _{j\rightarrow \infty }\Vert f^j_k \circ \phi ^j_k-f^j_{k-1}\Vert _{\mathcal {F}}^2 \end{aligned}$$

for every $k=1,\dots ,K$. To this end, we take into account the decomposition

$$\begin{aligned} f^j_k\circ \phi ^j_k-f_k\circ \phi _k&= (f^j_k\circ \phi ^j_k -f_k\circ \phi ^j_k)\\&\quad +(f_k\circ \phi ^j_k-f_k\circ \phi _k)\,. \end{aligned}$$

The second term is estimated as in the proof of (16). Thus, it remains to consider the convergence properties of the first term. For a test function $g\in \mathcal {F}$, we obtain using the transformation rule

$$\begin{aligned}&\int _\Omega (f^j_k\circ \phi ^j_k-f_k \circ \phi ^j_k)\cdot g\,\mathrm {d}x\\&\quad = \int _\Omega (f^j_k-f_k) \cdot (g(\det (D\phi ^j_k))^{-1})\circ (\phi ^j_k)^{-1}\,\mathrm {d}x\,. \end{aligned}$$

The right hand side converges to 0 due to the convergence $(\det (D\phi ^j_k))^{-1}\circ (\phi ^j_k)^{-1}\rightarrow \det (D\phi _k))^{-1}\circ \phi _k^{-1}$ in $L^\infty (\Omega )$ and $f_k^j \rightharpoonup f_k$ in $\mathcal {F}$ for $j\rightarrow \infty$. Thus, $f_k^j\circ \phi _k^j\rightharpoonup f_k\circ \phi _k$ for $j\rightarrow \infty$, which together with the lower semicontinuity of the $L^2$-norm proves (21). Altogether, we observe that $\mathbf {E}^K[\mathbf {f}]\le \mathbf {E}^{K,D} [\mathbf {f},\varvec{\Phi }]\le \liminf \limits _{j\rightarrow \infty } \mathbf {E}^{K,D}[\mathbf {f}^j,\varvec{\Phi }^j]\,.$ $\square$

## Convergence of Discrete Geodesic Paths

In this section, we provide a precise statement of the Mosco convergence for $K\rightarrow \infty$ of a suitable temporal extension of the time discrete path energy $\mathbf {E}^K$ in the deep metamorphosis model to the time continuous path energy $\mathcal {E}$ introduced in Definition 1. Furthermore, the convergence of time discrete geodesics to time continuous geodesic paths is established, which in particular implies the existence of time continuous geodesics in the deep feature metamorphosis model with an anisotropic regularizer.

We recall the definition of Mosco convergence [19], which can be seen as a modification of $\Gamma$–convergence. For further details, we refer the reader to [8].

### Definition 3

Let *X* be a Banach space. Consider functionals $\{\mathcal {E}^K\}_{K \in \mathbb {N}}$ and $\mathcal {E}$ from *X* to $\overline{\mathbb {R}}$ that satisfy

(i) for every sequence $\{x^K\}_{K\in \mathbb {N}}\subset X$ with $x^K\rightharpoonup x \in X$ the estimate $\liminf _{K\rightarrow \infty }{\mathcal {E}^K}[x^K]\ge \mathcal {E}[x]$ holds true (“$\liminf$–inequality”),
(ii) for every $x \in X$ there exists a recovery sequence $\{x^K\}_{K\in \mathbb {N}}\subset X$ satisfying $x^K\rightarrow x$ in *X* such that the estimate $\limsup _{K\rightarrow \infty }{\mathcal {E}^K}[x^K]\le \mathcal {E}[x]$ is valid.

Then, $\{\mathcal {E}_K\}_{K \in \mathbb {N}}$ converges to $\mathcal {E}$ in the sense of Mosco.

In what follows, we define temporal extensions of all relevant quantities required for the statement of the Mosco convergence. We remark that this construction is similar to [4, 13], where further details can be found.

To ensure that the involved deformations are diffeomorphisms and to avoid the interpenetration of matter along the morphing sequence, we replace in the definition of $\mathcal {A}$ the positivity constraint for the determinant by the stronger condition $\det (D\phi )\ge \varepsilon$ for a fixed (small) $\varepsilon >0$ as already proposed in [13]. Note that all existence results from the previous section remain valid for this modified definition.

For $K\in \mathbb {N}$, let $\varvec{\Phi }^K=(\phi _1^K,\ldots ,\phi _K^K)\in \mathcal {A}^K$ be the vector of deformations associated with a vector of features $\mathbf {f}^K=(f_0^K,\ldots ,f_K^K)\in \mathcal {F}^{K+1}$. In particular, if $\varvec{\Phi }^K$ is optimal, then $\mathbf {E}^K[\mathbf {f}^K]=\mathbf {E}^{K,D}[\mathbf {f}^K,\varvec{\Phi }^K]$. For $k=1,\ldots ,K$, we define for $t_k^K:=\frac{k}{K}$, $t\in [t_{k-1}^K,t_k^K]$, and $x\in \Omega$ the *discrete transport map*

22 $$\begin{aligned} y_k^K(t,x)=x+(t-t^K_{k-1})K(\phi _k^K(x)-x)\,. \end{aligned}$$

Note that $y_k^K(t^K_{k-1},x)=x$ and $y_k^K(t_k^K,x)=\phi _k^K(x)$. Following [7, Chapter 5], the condition

$$\begin{aligned} \max _{k=1,\ldots ,K}\Vert D\phi _k^K-{\mathbb {1}}\Vert _{C^0({\overline{\Omega }})}<1 \end{aligned}$$

implies that $y_k^K(t,\cdot )$ is invertible, which follows for *K* large enough from the $\liminf$-part of the proof of Theorem 2 below, and we denote the inverse by $x_k^K(t,\cdot )$. In this case, we consider the *feature extension operator* ${\mathcal {F}^K}[\mathbf {f}^K,\varvec{\Phi }^K]\in L^2([0,1]\times \mathcal {F})$ for $t\in [t^K_{k-1},t_k^K]$ by

$$\begin{aligned}&{\mathcal {F}^K}[\mathbf {f}^K,\varvec{\Phi }^K](t,x)\\&\quad :=\Big (f_{k-1}^K+K(t-t^K_{k-1}) (f_k^K\circ \phi _k^K-f_{k-1}^K)\Big )(x_k^K(t,x))\,, \end{aligned}$$

to define an extension ${\mathcal {E}^K}:L^2([0,1]\times \mathcal {F})\rightarrow [0,\infty ]$ of the discrete path energy $\mathbf {E}^{K,D}$, where

$$\begin{aligned}&\mathcal {E}^{K}[f]= \inf _{\overline{\varvec{\Phi }}^K\in \mathcal {A}^K} \left\{ {\mathbf {E}^{K,D}}[\mathbf {f}^K,\overline{\varvec{\Phi }}^K]: {\mathcal {F}^K}[\mathbf {f}^K,\overline{\varvec{\Phi }}^K]=f\right\} \end{aligned}$$

if there exist $\mathbf {f}^K\in \mathcal {F}^{K+1}$ and $\varvec{\Phi }^K\in \mathcal {A}^K$ such that $f={\mathcal {F}^K}(\mathbf {f}^K,\varvec{\Phi }^K)$, else we set ${\mathcal {E}^{K}}[f]=\infty$.

We can now state the main theorem of this section:

### Theorem 2

(Mosco convergence of the discrete path energies) Let (W1)–(W3) and (A1)–(A3) be satisfied. Then, the time discrete path energy $\{\mathcal {E}^K\}_{K\in \mathbb {N}}$ converges to $\mathcal {E}$ in the sense of Mosco in the $L^2([0,1]\times \mathcal {F})$-topology for $K\rightarrow \infty$.

### Proof

The proof follows the structure of the Mosco convergence proof [13, Theorem 5.2 & Theorem 5.4] with adaptations required due to the incorporation of the anisotropy. Furthermore, in our case images are not pointwise maps into a general Hadamard manifold but rather maps into some Euclidean space. To keep the exposition compact, we focus here on these adaptations. To facilitate reading, we give an overview of the general structure of the proof, which retrieves the overview of the proof structure in [13]. Many of the technical arguments already appeared in the proof of existence of discrete geodesics in Sect. 3 and were given there in full detail. Thus, we keep these arguments brief here.

(i) $$\liminf $$ **-inequality.**

- The *identification of the image (feature) and deformation vectors* is unaltered compared to the proof in [13]. Indeed, one obtains that the sequence $f^K$ of feature maps with uniformly bounded energy converges weakly to a feature map $f\in L^2([0,1],\mathcal {F})$ with finite energy. In fact, $\mathbf {f}^K$ and the optimal $\overline{\varvec{\Phi }}^K$ can be retrieved from $f^K$, where the existence of $\overline{\varvec{\Phi }}^K$ follows as in [13, Lemma 5.1].
- The verification of the *lower semi-continuity of the weak material derivative* in the sense
  $$\begin{aligned} \Vert z\Vert _{L^2([0,1]\times \Omega )}^2 \le \liminf _{K\rightarrow \infty }K\sum _{k=1}^K\Vert f_{k-1}^K -f_k^K\circ \overline{\phi }_k^K\Vert _{L^2(\Omega )}^2\,. \end{aligned}$$
  for *z* being the weak limit of $z^K$ in $L^2((0,1) \times \Omega )$ with
  $$\begin{aligned} z^K\big \vert _{[t_{k-1}^K,t_k^K)}:=K|f_{k-1}^K(x_k^K)-f_k^K\circ \overline{\phi }_k^K(x_k^K)| \end{aligned}$$
  is identical to the corresponding reasoning in [13]. At this point, we also observe that the velocity field $$w^K$$ with $$w^K:=w_k^K:=K(\overline{\phi }_k^K-{\mathbb {1}})$$ on $$[t_{k-1}^K,t_k^K)$$ is uniformly bounded in $$L^2((0,1),\mathcal {V})$$ and thus converges weakly in $$L^2((0,1),\mathcal {V})$$ to some limit velocity field *v*. This fact is again proved following the corresponding reasoning as in [13].
- Also the *verification of the admissibility of the limit*, i.e., $(v,z)\in \mathcal {C}(f)$, stays unaltered compared to [13]. To this end, one shows that the discrete flow $\psi ^K$ associated with the motion field $v^K(t,x):=K(\overline{\phi }_k^K-{\mathbb {1}})(x_k^K(t,x))$ for $t\in [t_{k-1}^K,t_k^K)$ uniformly converges in $C^{0,\alpha }([0,1],C^{1,\alpha }({\overline{\Omega }}))$ for a suitable constant $\alpha >0$ to the continuous flow induced by the velocity *v*. Moreover, the variational inequality for the material derivative holds true in the limit.
- In the final step, the *lower semi-continuity of the viscous dissipation* has to be shown, i.e.,
  $$\begin{aligned}&\int _0^1\int _\Omega L[a[\mathcal {P}[f]],v,v]\,\mathrm {d}x\,\mathrm {d}t\\&\quad \le \liminf _{K\rightarrow \infty }K\sum _{k=1}^K\int _\Omega a[\mathcal {P}[f_k^K]]\mathrm {W}(D{\overline{\phi }}_k^K)+\gamma \vert D^m{\overline{\phi }}_k^K\vert ^2\,\mathrm {d}x\,. \end{aligned}$$
  Therefore, we define $a^K,\overline{a}^K\in L^\infty ((0,1)\times \Omega ,\mathbb {R}^+)$ via $\overline{a}^K\big \vert _{[t_{k-1}^K,t_k^K)}:=a[\mathcal {P}[f_k^K]]$ and $a^K:=a[\mathcal {P}[f^K]]$. We have to show in addition to [13] that $\overline{a}^K$ converges strongly to $a:=a[\mathcal {P}[f]]$ in $L^\infty ((0,1)\times \Omega )$. To this end, we first use the uniform boundedness of $f^K$ in $L^\infty ([0,1],\mathcal {F})$, an approximation argument and (A3) to show the convergence $\overline{a}^K-a^K\rightarrow 0$ in $L^\infty ((0,1)\times \Omega )$ for $K\rightarrow \infty$. It remains to verify $a^K\rightarrow a$ in $L^\infty ((0,1)\times \Omega )$ for $K\rightarrow \infty$. The variational inequality
  $$\begin{aligned} \vert f^K(t,\psi _t^K(x))-f^K(s,\psi _s^K(x))\vert \le \int _s^t z^K(r,\psi _r^K(x))\,\mathrm {d}r \end{aligned}$$

implies $f^K(t)\rightharpoonup f(t)$ in $\mathcal {F}$ for every $t\in [0,1]$ using similar arguments as in step (iv) of [4, Theorem 4.1], which leads to $a^K(t,\cdot )\rightarrow a(t,\cdot )$ in $L^\infty (\Omega )$ using (A2). We are left to show

$$\begin{aligned} \Vert a^K(t+\tau ,\cdot )-a^K(t,\cdot )\Vert _{\mathcal {F}}\rightarrow 0 \end{aligned}$$

uniformly in *K* and *t* as $\tau \rightarrow 0$, which follows by (A3) from $\Vert f^K(t+\tau ,\cdot )-f^K(t,\cdot )\Vert _{\mathcal {F}}\rightarrow 0$. This equicontinuity in time is a consequence of the variational inequality, the uniform boundedness of $z^K$ in $L^2((0,1)\times \Omega )$, the uniform boundedness of $\psi ^K$ and $(\psi ^K)^{-1}$ in $C^{0,\frac{1}{2}}([0,1],C^{1,\alpha }({\overline{\Omega }}))$ for a suitable $\alpha >0$, and an approximation argument for $f^K$. Then, the actual lower semicontinuity is verified using a Taylor expansion of $\mathrm {W}$ based on (W3) to relate the energy density *W* with *L*, where the accumulated remainder is of order $K^{-\frac{1}{2}}$.

(ii) **Recovery sequence.** Before constructing the recovery sequence, we note that the infimum in (6) is actually attained with an associated pair $(v,z)\in \mathcal {C}(f)$, which follows from [13, Proposition 5.3] together with Remark 1.

- To *construct the recovery sequence*, one considers the above pair $(v,z)\in \mathcal {C}(f)$ with an associated flow $\psi$ and defines $\phi _k^K(x):=\psi _{t_{k-1}^K,t_k^K}(x)$, $f_k^K(x):=f(t_k^K,x)$, and $a_k^K=a[\mathcal {P}[f_k^K]]$ for $k=1,\ldots ,K$, where
  $$\psi _{a,b}(\cdot )=\psi (b,\psi ^{-1}(a,\cdot ))$$, for $$a,b\in [0,1]$$.
- Next, the *identification of the recovery sequence limit* is done, i.e., one can show that the extension $f^K:={\mathcal {F}^K}[\mathbf {f}^K,\varvec{\Phi }^K]$ of the time discrete feature vectors $\mathbf {f}^K=(f_0,\dots ,f_K)$ converges to $f$ in $L^2([0,1],\mathcal {F})$. To this end, the discrete flow $\psi ^K$ associated with the time discrete family of deformations $\varvec{\Phi }^K$ is defined in the same way as in the proof of the $\liminf$–inequality. Following [13], the convergence $f^K\rightarrow f$ is implied by the variational inequality and the convergence of $\psi ^K$ to the time continuous flow $\psi$ associated with *v* in $C^{0,\alpha }([0,1],C^{1,\alpha }({\overline{\Omega }}))$ for a suitable $\alpha >0$.
- Furthermore, we have to *verify the* $\limsup$-*inequality.* The leading order term of a Taylor expansion of the *k*-th component of the discrete path energy
  $$\begin{aligned} \int _\Omega a_k^K\mathrm {W}(D\phi _k^K)+\gamma |D^m\phi _k^K|^2\,\mathrm {d}x \end{aligned}$$
  is given by $K^{-2}\int _\Omega L[a_k^K,w_k^K,w_k^K]\,\mathrm {d}x$, where $w_k^K:=K(\phi _k^K-{\mathbb {1}})$. The remainder is of higher order following the argumentation in [13]. Using Jensen’s inequality and  with $\psi _k^K(t,x):=\psi _{t_{k-1}^K,t}(x)$ we obtain

for $\overline{a}^K$ defined as before. Following [13], we can replace $\psi _k^K(t,\cdot )$ by the identity in the limit $K\rightarrow \infty$. We argue analogously as in the case of the $\liminf$–inequality to show $\overline{a}^K\rightarrow a$ in $L^{\infty }((0,1)\times \Omega )$. Thus, we obtain

$$\begin{aligned}&\limsup _{k\rightarrow \infty }K\sum _{k=1}^K\int _\Omega a_k^K \mathrm {W}(D\phi _k^K)+\gamma |D^m\phi _k^K|^2\,\mathrm {d}x\\&\quad \int _0^1\int _\Omega L[a(t,x),v(t,x),v(t,x)]\,\mathrm {d}x\,\mathrm {d}t\,. \end{aligned}$$

Finally, the estimate

$$\begin{aligned} \limsup _{k\rightarrow \infty } K \int _\Omega \!\! \vert f_{k-1}^K\!-f_k^K\circ \phi _k^K\vert ^2\,\mathrm {d}x \le \int _0^1\!\!\int _\Omega \!\! z^2(t,x)\,\mathrm {d}x\,\mathrm {d}t \end{aligned}$$

follows via another application of Jensen’s inequality as in [13]. $\square$

This theorem implies the convergence and existence of geodesic paths for the (time continuous) deep feature space metamorphosis model in the following sense:

### Theorem 3

(Convergence of discrete geodesics) Suppose that the assumptions (W1)–(W3) and (A1)–(A3) hold true. Let $f_A,f_B\in \mathcal {F}$ be fixed. For $K\in \mathbb {N}$ sufficiently large let $f^K$ be a minimizer of $\mathcal {E}^K$ subject to $f^K(0)=f_A$ and $f^K(1)=f_B$. Then, a subsequence of $\{f^K\}_{K\in \mathbb {N}}$ weakly converges in $L^2([0,1]\times \mathcal {F})$ to a minimizer of the continuous path energy $\mathcal {E}$ as $K\rightarrow \infty$. Finally, the associated sequence of discrete path energies converges to the minimal continuous path energy.

### Proof

The proof is analogous to the proof of [13, Theorem 5.5]. $\square$

## Fully Discrete Model in Feature Space

In this section, we present the fully discrete deep feature space metamorphosis model on the image domain $\Omega =[0,1]^2$. We use bold face letters to differentiate discrete feature maps, images, and deformations (also considered as vectors) from their continuous counterparts. For $\text {for }M,N\ge 3$, we define the computational domain and its boundary as follows:

$$\begin{aligned} \Omega _{{\tiny M\!N}}&=\left\{ \frac{0}{M-1},\ldots ,\frac{M-1}{M-1}\right\} \times \left\{ \frac{0}{N-1},\ldots ,\frac{N-1}{N-1}\right\} \,,\\ \partial \Omega _{{\tiny M\!N}}&=\Omega _{{\tiny M\!N}}\backslash \left\{ \frac{1}{M-1},\ldots ,\frac{M-2}{M-1}\right\} \times \left\{ \frac{1}{N-1}, \ldots ,\frac{N-2}{N-1}\right\} \,. \end{aligned}$$

We define the discrete $L^p$-norm of a discrete feature map $\mathbf {f}$ as

$$\begin{aligned} \left\| \mathbf {f}\right\| _{L^p(\Omega _{{\tiny M\!N}})}^p =\frac{1}{MN}\sum _{(i,j)\in \Omega _{{\tiny M\!N}}}\Vert \mathbf {f}(i,j)\Vert _2^p, \end{aligned}$$

and the set of admissible deformations is given by

$$\begin{aligned} \mathcal {A}_{{\tiny M\!N}}&=\Big \{\varvec{\phi }:\Omega _{{\tiny M\!N}}\rightarrow \Omega _{{\tiny M\!N}}: \\&\varvec{\phi }={\mathbb {1}}\text { on }\partial \Omega _{{\tiny M\!N}},\ {\det (\mathbf {\nabla }_{\tiny M\!N}\varvec{\phi })}>0\Big \}\,. \end{aligned}$$

Furthermore, the discrete Jacobian operator $\mathbf {\nabla }_{\tiny M\!N}$ of $\varvec{\phi }$ at $(i,j)\in \Omega _{{\tiny M\!N}}$ is defined as the forward finite difference operator with Neumann boundary conditions. To further stabilize the computation, the Jacobian operator applied to the features is approximated using a Sobel filter. The discrete image space and the discrete feature space are given by $\mathcal {I}_{{\tiny M\!N}}=\{\mathbf {u}:\Omega _{{\tiny M\!N}}\rightarrow \mathbb {R}^3\}$ and $\mathcal {F}_{{\tiny M\!N}}=\{\mathbf {f}:\Omega _{{\tiny M\!N}}\rightarrow \mathbb {R}^{3+C}\}$, respectively.

A numerically reasonable approximation of the spatial warping operator $\mathbf {T}$, which approximates the pullback of a feature channel $\mathbf {f}\circ \varvec{\phi }$ at a point $(k,l)\in \Omega _{{\tiny M\!N}}$, is given by

$$\begin{aligned}&\mathbf {T}[\mathbf {f},\varvec{\phi }](k,l) \\&\quad = \sum _{(i,j)\in \Omega _{{\tiny M\!N}}}\mathbf {s} (\varvec{\phi }_{1}(k,l)-i)\mathbf {s} (\varvec{\phi }_{2}(k,l)-j)\mathbf {f}(i,j)\,, \end{aligned}$$

where $\mathbf {s}$ is the third order B-spline interpolation kernel. Then, the fully discrete mismatch functional $\mathbf {D}_{{\tiny M\!N}}$ that approximates $\int _\Omega |\tilde{f}\circ \phi -f|^2\,\mathrm {d}x$ reads as

$$\begin{aligned}&\mathbf {D}_{{\tiny M\!N}}[\mathbf {f},{\tilde{\mathbf {f}}}, \varvec{\phi }]\\&\quad = \frac{1}{2(3+C)}\sum _{c=1}^{3+C}\left\| \mathbf {T}[\tilde{\mathbf {f}}^c,\varvec{\phi }]- \mathbf {f}^c\right\| _{L^2(\Omega _{{\tiny M\!N}})}^2\,. \end{aligned}$$

Likewise, the lower order anisotropic regularization functional $\int _\Omega a\mathrm {W}(D\phi )\,\mathrm {d}x$ is discretized as follows:

$$\begin{aligned} \mathbf {R}_{{\tiny M\!N}}[\varvec{\phi },\mathbf {a}] =\Vert \mathbf {a}\mathrm {W}({\mathbf {\nabla }_{\tiny M\!N}} \varvec{\phi })\Vert _{L^1(\Omega _{{\tiny M\!N}})}\,. \end{aligned}$$

For simplicity, we neglect the $H^m$-seminorm of the deformations. In the spatially continuous context of the above convergence proof the compactness induced by the $H^m$-seminorm turned out to be indispensable. In the case of the spatial discretization, the grid-dependent regularity is ensured by the use of cubic B-splines.

In summary, the fully discrete path energy in the deep metamorphosis model for a $(K+1)$-tuple $(\mathbf {f}_k)_{k=0}^K$ of discrete feature maps, a *K*-tuple $(\varvec{\phi }_k)_{k=1}^K$ of discrete deformations, and a *K*-tuple $(\mathbf {a}_k)_{k=1}^K$ of discrete anisotropies reads as

$$\begin{aligned}&{\mathbf {E}_{\tiny M\!N}^{K}}[(\mathbf {f}_k)_{k=0}^K, (\varvec{\phi }_k)_{k=1}^K,(\mathbf {a}_k)_{k=1}^K]\\&\quad = K\sum _{k=1}^K\mathbf {R}_{{\tiny M\!N}}[\varvec{\phi }_k, \mathbf {a}_k]+\frac{1}{\delta }\mathbf {D}_{{\tiny M\!N}} [\mathbf {f}_{k-1},\mathbf {f}_k,\varvec{\phi }_k]\,. \end{aligned}$$

Finally, a discrete geodesic path $(\mathbf {f}_k)_{k=0}^{K}$ in feature space on a specific multiscale level of a feature hierarchy is a minimizer of $\mathbf {E}_{\tiny M\!N}^{K}$ subject to given discrete boundary data $\mathbf {f}_0=\mathbf {f}_A$ and $\mathbf {f}_K=\mathbf {f}_B$. Here, $\mathbf {f}_A=(\eta \mathbf {u}_A,\mathbf {F}_{\tiny M\!N}(\mathbf {u}_A))$ and $\mathbf {f}_B=(\eta \mathbf {u}_B,\mathbf {F}_{\tiny M\!N}(\mathbf {u}_B))$, where $\mathbf {F}_{{\tiny M\!N}}:\mathcal {I}_{{\tiny M\!N}}\rightarrow \{\mathbf {f}: \Omega _{{\tiny M\!N}}\rightarrow \mathbb {R}^C\}$ denotes the fully discrete feature extraction operator.

Simple RGB Model As a first model, we consider the simple image intensity-based feature space with $C=0$, in which the feature space $\mathcal {F}_{{\tiny M\!N}}$ coincides with the space of RGB images $\mathcal {I}_{{\tiny M\!N}}$. Since a direct computation of the deformations on the full grid is numerically instable, we incorporate a multilevel scheme. Initially, we start on the coarsest computational domain of size $M_{\mathrm {init}}\times N_{\mathrm {init}}$ with $M_{\mathrm {init}}=2^{-(L-1)}M$ and $N_{\mathrm {init}}=2^{-(L-1)}N$ for a given $L>0$ and compute a time discrete geodesic sequence for suitably resized input images  $\mathbf {u}_A,\mathbf {u}_B$. Then, in subsequent prolongation steps, the width and the height of the computational domain are successively doubled and the initial deformations and images are obtained via a bilinear interpolation of the preceding coarse scale solutions.

Deep Feature Space In the second model, image features are extracted using the prominent VGG network with 19 layers as presented in [28] to incorporate semantic information in image morphing. The VGG network is particularly designed for localization and classification of objects in natural images and thus the feature decomposition of images is well-suited for semantic matching. The building blocks of this network are convolutional layers with subsequent ReLU nonlinear activation functions and max pooling layers. Here, the max pooling layers canonically yield a multiscale semantic decomposition of images.

For a given grid $\Omega _{{\tiny M\!N}}$, the discrete feature maps of the fixed discrete input images $\mathbf {u}_A$ and $\mathbf {u}_B$ are $\mathbf {F}_{{\tiny M\!N}}[\mathbf {u}_A]$ and $\mathbf {F}_{{\tiny M\!N}}[\mathbf {u}_B]$, where the operator $\mathbf {F}_{{\tiny M\!N}}$ is the response of the VGG network up to the layer as shown in Table 1. The discrete images $\mathbf {u}_A$ and $\mathbf {u}_B$ are downsampled to match the corresponding grid size *MN* via a bilinear interpolation. In contrast to the simple RGB model, only the deformations are prolongated via a bilinear interpolation in the multilevel approach since successive features on different multilevels are not necessarily related. To stabilize the optimization, the features on each multilevel are first optimized using the prolongated deformations.

**Table 1:** Multiscale decomposition of the VGG network used for the discrete feature extraction operator $\mathbf {F}_{{\tiny M\!N}}$

$M\times N$	Layer	*C*
$512\times 512$	$\mathrm {conv}_{1,2}$	64
$256\times 256$	$\mathrm {conv}_{2,2}$	128
$128\times 128$	$\mathrm {conv}_{3,4}$	256
$64\times 64$	$\mathrm {conv}_{4,4}$	512
$32\times 32$	$\mathrm {conv}_{5,4}$	512

**Table 2:** The parameter values for all examples

Parameter	RGB	Deep
*K*	15	
$\delta$	1	
*L*	5	
$\beta$	$\frac{1}{\sqrt{2}}$	
*J*	250	
$\sigma$	0.5	
$\rho$	2	
$\xi _1$	1000	
$\xi _2$	$10^{-6}$	
$\mu$	0.025	0.002
$\lambda$	0.1	0.002
$\eta$		$10^{-6}$

**Fig. 1:**
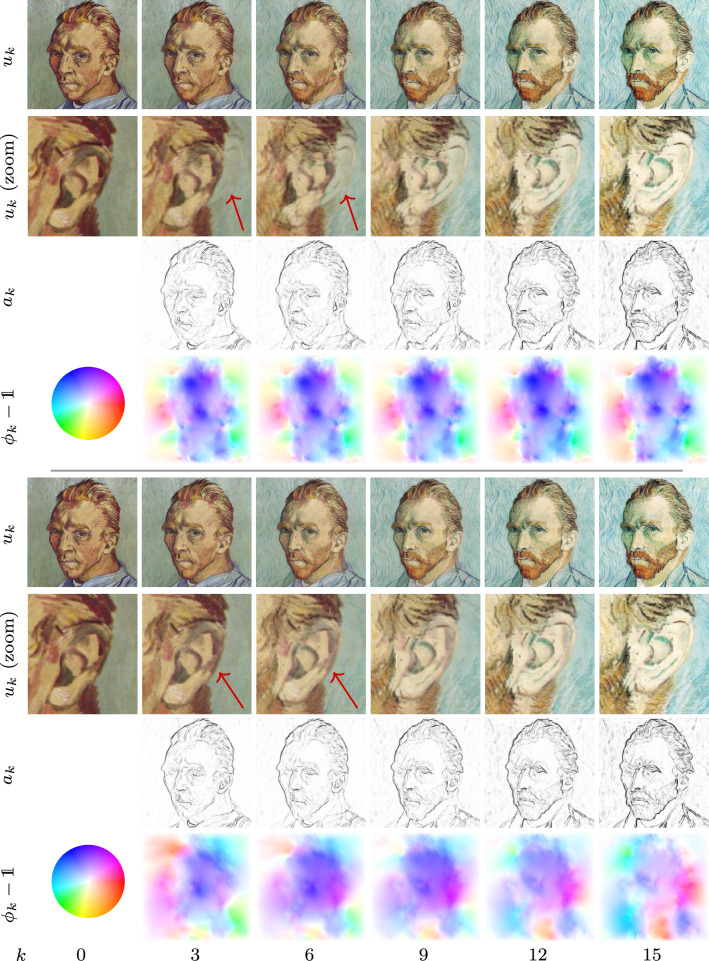
Time discrete geodesic sequences of self-portraits by van Gogh for the RGB feature (first row) and deep feature model (fifth row) along with a zoom of the ear region with magnification factor 4 (second/sixth row) and the associated sequences of anisotropy weights (third/seventh row) and color-coded displacement fields $\phi _k-{\mathbb {1}}$ (fourth/eighth row). Note that the intensity-based approach leads to blending artifacts indicated by the arrows, which are resolved in the deep metamorphosis model

**Fig. 2:**
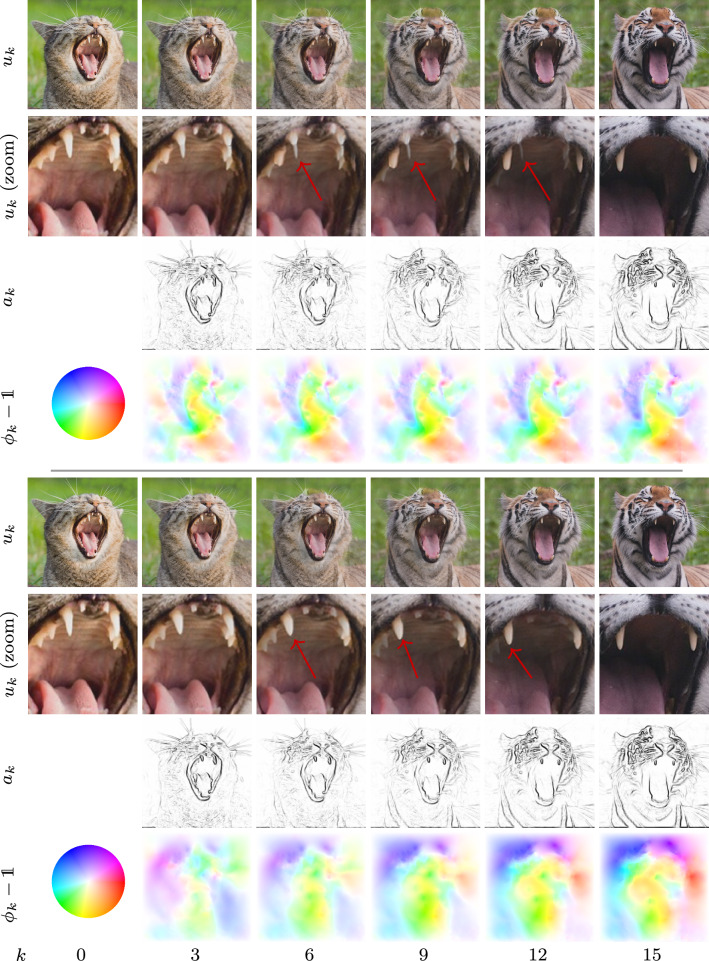
Time discrete geodesic sequences of animal photos for the RGB feature (first row) and deep feature model (fifth row) along with a zoom of the mouth region with magnification factor 4 (second/sixth row) and the associated sequences of anisotropy weights (third/seventh row) and color-coded displacement fields $\phi _k-{\mathbb {1}}$ (fourth/eighth row). Note that the novel deep feature-based model has significantly less blending artifacts as indicated by the arrows

**Fig. 3:**
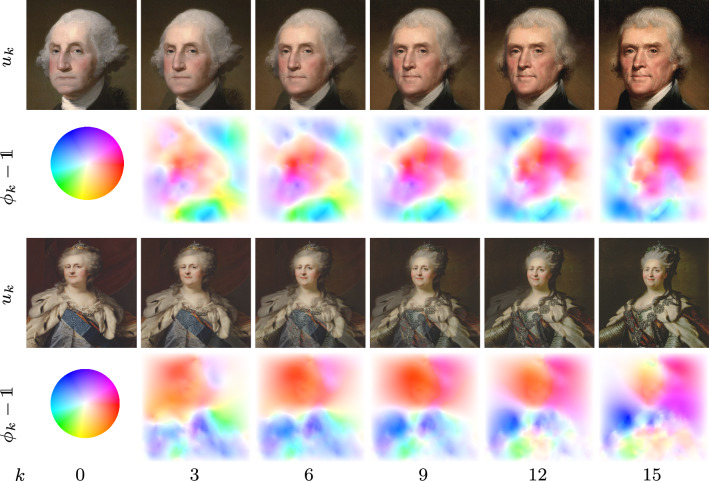
Pairs of time discrete geodesic paths using the deep feature model and corresponding color-coded displacement fields for paintings of US presidents (first/second row) as well as for paintings of Catherine the Great (third/forth row)

### Numerical Optimization

In what follows, we present the numerical optimization scheme to compute geodesics for the fully discrete deep feature metamorphosis model. Here, we use a variant of the inertial proximal alternating linearized minimization algorithm (iPALM, [24]). Several numerical experiments indicate that a direct gradient based minimization of the data mismatch term $\mathbf {D}_{{\tiny M\!N}}$ with respect to the deformations is challenging due to the sensitivity of the warping operator to small perturbations of the deformations. Thus, to enhance the stability of the algorithm the warping operator is linearized w.r.t. the deformation at ${\tilde{\varvec{\phi }}}\in \mathcal {A}_{{\tiny M\!N}}$, which is chosen as the deformation of the previous iteration step in the algorithm. To further improve the stability of the algorithm, the linearization is based on the gradient $\Lambda _c(\mathbf {f},{\tilde{\mathbf {f}}}, {\tilde{\varvec{\phi }}})=\frac{1}{2} ({\mathbf {\nabla }_{\tiny M\!N}}\mathbf {T}[{\tilde{\mathbf {f}}}^c, {\tilde{\varvec{\phi }}}]+{\mathbf {\nabla }_{\tiny M\!N}}\mathbf {f}^c)$, which yields the modified mismatch energy

$$\begin{aligned}&{\widetilde{\mathbf {D}}}_{{\tiny M\!N}}[\mathbf {f}, {\tilde{\mathbf {f}}},\varvec{\phi },{\tilde{\varvec{\phi }}}] =\frac{1}{2(3+C)}\sum _{c=1}^{3+C}\bigg \Vert \mathbf {T}[{\tilde{\mathbf {f}}}^c,{\tilde{\varvec{\phi }}}]\\&\qquad \qquad \quad \qquad +\left\langle \Lambda _c (\mathbf {f},{\tilde{\mathbf {f}}},{\tilde{\varvec{\phi }}}), \varvec{\phi }-{\tilde{\varvec{\phi }}}\right\rangle - \mathbf {f}^c\bigg \Vert _{L^2(\Omega _{{\tiny M\!N}})}^2\,. \end{aligned}$$

**Fig. 4:**
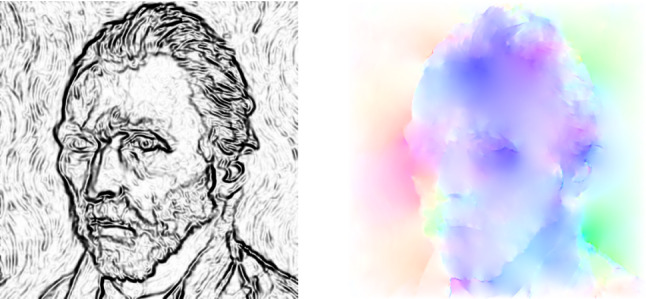
Visualization of the anisotropy in RGB model for a significant smaller value $\xi _1=200$ compared to Fig. 1: anisotropy weights (left) and color-coded displacement fields (right) for $k=12$

**Fig. 5:**
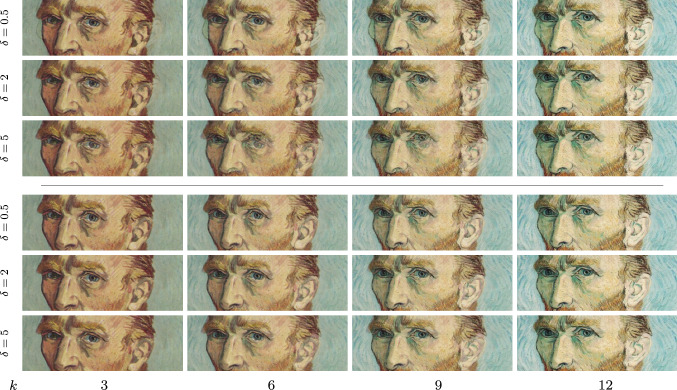
Variation of the parameter $\delta$ for RGB model (first to third row) and deep feature model (fourth to sixth row)

The mismatch energy can be efficiently minimized incorporating a proximal mapping, which is defined for a function $\mathbf {f}:\Omega _{{\tiny M\!N}}\rightarrow (-\infty ,\infty ]$ for $\tau >0$ as follows:

$$\begin{aligned} {\text {prox}}_\tau ^\mathbf {f}(i):= \mathop {\mathrm{argmin}}_{j:\Omega _{{\tiny M\!N}}\rightarrow (-\infty ,\infty ]}\frac{\tau }{2}\left\| i-j\right\| _{L^2(\Omega _{{\tiny M\!N}})}^2+\mathbf {f}(j)\,. \end{aligned}$$

The proximal operator with respect to the deformation $\varvec{\phi }$ for a fixed $\tau >0$ is given by

$$\begin{aligned}&{\text {prox}}_{\tau }^{\frac{K}{\delta }{\widetilde{\mathbf {D}}}_{{\tiny M\!N}}} [\varvec{\phi }]\\&\quad = \left( {\mathbb {1}}+\frac{K}{\tau \delta (3+C)}\sum _{c=1}^{3+C}\Lambda _c (\mathbf {f},{\tilde{\mathbf {f}}},{\tilde{\varvec{\phi }}}) \Lambda _c(\mathbf {f},{\tilde{\mathbf {f}}},{\tilde{\varvec{\phi }}})^\top \right) ^{-1} \\&\Bigg (\varvec{\phi }-\frac{K}{\tau \delta (3+C)} \sum _{c=1}^{3+C}\Big (\Lambda _c(\mathbf {f},{\tilde{\mathbf {f}}}, {\tilde{\varvec{\phi }}})\mathbf {T}[{\tilde{\mathbf {f}}}^c, {\tilde{\varvec{\phi }}}]\\&-\Lambda _c(\mathbf {f},{\tilde{\mathbf {f}}}, {\tilde{\varvec{\phi }}})\Lambda _c(\mathbf {f}, {\tilde{\mathbf {f}}},{\tilde{\varvec{\phi }}})^\top {\tilde{\varvec{\phi }}}-\Lambda _c(\mathbf {f},{\tilde{\mathbf {f}}}, {\tilde{\varvec{\phi }}})\mathbf {f}^c\Big )\Bigg )\,, \end{aligned}$$

where the function values on $\partial \Omega _{{\tiny M\!N}}$ remain unchanged.


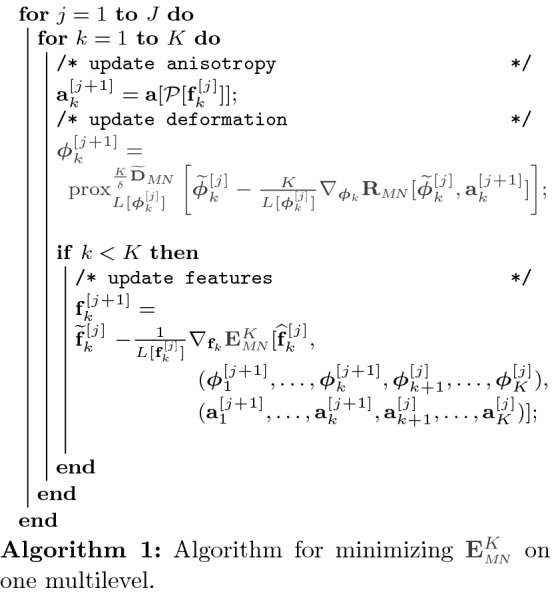


Algorithm 1 summarizes the iteration steps for the minimization of the fully discrete path energy $\mathbf {E}^K_{{\tiny M\!N}}$, where for a specific optimization variable $\mathbf {f}$ the extrapolation with $\beta >0$ of the $k^{th}$ path element in the $j^{th}$ iteration step reads as follows:

$$\begin{aligned}&\widetilde{\mathbf {f}}_k^{[j]}=\mathbf {f}_k^{[j]} +\beta (\mathbf {f}_k^{[j]}-\mathbf {f}_k^{[j-1]}),\\&\widehat{\mathbf {f}}_k^{[j]}=(\mathbf {f}_0^{[j+1]}, \ldots ,\mathbf {f}_{k-1}^{[j+1]}, \widetilde{\mathbf {f}}_k^{[j]},\mathbf {f}_{k+1}^{[j]}, \ldots ,\mathbf {f}_{K}^{[j]})\,. \end{aligned}$$

Here, we use the notation $L[\mathbf {f}]$ for the Lipschitz constant of the function $\mathbf {f}$, which is determined by backtracking.

## Numerical Results

In this section, numerical results for both the RGB and the deep feature model are shown. All parameters used in the computation are specified in Table 2.

Figure 1 depicts the geodesic sequences for two self-portraits by van Gogh^1^ ($M \times N= 496 \times 496$) for $k\in \{0,3,6,9,12,15\}$ obtained with the RGB model (first row) and the deep feature model (fifth row). The superiority of the deep model compared to the simple RGB model is exemplarily visualized by the zoom (magnification factor 4) of the ear region depicted in the second and sixth row. The remaining rows contain the corresponding sequences of anisotropy operators (third/seventh row) and color-coded displacement fields (fourth/eighth row), where the hue refers to the direction of the displacements and the intensity is proportional to its norm as indicated by the leftmost color wheel. Figure 2 presents analogous results for two photos of animals^2^ for $M\times N=512\times 512$ with a zoom on the mouth region. Note that the deep model is capable of accurately deforming the carnassial teeth.

Figure 3 shows results of the deep feature model for two paintings of US presidents^3^ and two portraits of Catherine the Great.^4^ In both cases, the input images have a resolution of $M\times N=512\times 512$.

Finally, we examine the effects of parameter changes of $\xi _1$ and $\delta$. Figure 4 visualizes the anisotropy weight and the deformation field in the RGB model for a $\xi _1$ value fostering a significantly stronger anisotropy implying much more pronounced jumps in the deformation field (compare with Fig. 1). In addition, Fig. 5 illustrates the dependency of the resulting morphing sequences on $\delta$ for the RGB model (first to third row) and the deep model (fourth to sixth row). As a result, smaller values of $\delta$ lead to less blending. Furthermore, the generated geodesic paths using deep features are more robust to changes of $\delta$ than the RGB model, which can, for instance, be seen in the cheek or in the eye regions.

In all numerical experiments, the displacement fields apparently evolve over time and the involved anisotropy promotes large deformation gradients in the proximity of image interfaces. These are indicated by the sharp interfaces in the color coding of the deformations. Both models fail to match image regions with no obvious correspondence of the input images, which can be seen on the cloth regions of the self-portraits, the presidents and the empress examples, as well as on parts of the body region and the background in the animal example, where blending artifacts occur. The deep feature model clearly outperforms the simple RGB model in regions where the semantic similarity is not reflected by the RGB color features such as the cheek and the ear in the van Gogh example as well as the teeth of the animals. Moreover, to compute a visually appealing time discrete geodesic sequence, a fourth color channel representing a manual segmentation of image regions and a color adaptation of the van Gogh self-portraits was required in [4]. This is obsolete in the proposed deep feature-based model due to the incorporation of semantic information.

## Footnotes

1 Public domain, https://commons.wikimedia.org/wiki/File:Vincent_Willem_van_Gogh_102.jpg; https://commons.wikimedia.org/wiki/File:SelbstPortrait_VG2.jpg.

2 First photo detail by Domenico Salvagnin (CC BY 2.0), https://commons.wikimedia.org/wiki/File:Yawn!!!_(331702223).jpg; second photo detail by Eric Kilby (CC BY-SA 2.0), https://commons.wikimedia.org/wiki/File:Panthera_tigris_-Franklin_Park_Zoo,_Massachusetts,_USA-8a_(2).jpg.

3 First painting by Gilbert Stuart (public domain), https://commons.wikimedia.org/wiki/File:Gilbert_Stuart_Williamstown_Portrait_of_George_Washington.jpg; second painting by Rembrandt Peale (public domain), https://commons.wikimedia.org/wiki/File:Thomas_Jefferson_by_Rembrandt_Peale,_1800.jpg.

4 Public domain, both portraits by J. B. Lampi https://commons.wikimedia.org/wiki/File:Catherine_II_by_J.B.Lampi_(Deutsches_Historisches_Museum).jpg; https://commons.wikimedia.org/wiki/File:Catherine_II_by_J.B.Lampi_(1780s,_Kunsthistorisches_Museum).jpg.
